# Radiological findings of denosumab treatment for giant cell tumours of bone

**DOI:** 10.1007/s00256-020-03449-1

**Published:** 2020-04-26

**Authors:** Kirsten van Langevelde, Catherine L. McCarthy

**Affiliations:** 1 Radiology Department, Nuffield Orthopaedic Centre, Oxford, OX3 7HE UK; 2 Radiology Department, Leiden University Medical Center, Leiden, The Netherlands

**Keywords:** Giant cell tumour of bone, Denosumab, MRI, CT, PET-CT, Imaging

## Abstract

Giant cell tumours of bone (GCTB) are benign giant cell-rich tumours typically occurring in the epi-metaphysis of skeletally mature patients. Despite their benign classification, GCTB may be locally aggressive with local recurrence as a challenging issue. Denosumab is a human monoclonal antibody that inhibits osteolysis via the RANK-RANK ligand pathway. There is currently no consensus on optimal treatment duration or imaging modality for monitoring patients on denosumab therapy. This review illustrates the radiological findings of GCTB on denosumab treatment seen on plain radiographs, CT, MRI, PET-CT and DEXA, with reference to the current literature. Recognizing imaging features indicative of a positive response to denosumab is important for therapeutic decision-making. Imaging findings with respect to duration of denosumab treatment, tumour upregulation during treatment, tumour recurrence and malignant transformation are discussed. The development of a sclerotic neocortex and varying degrees of matrix osteosclerosis are seen on plain radiographs. Reconstitution of subarticular bone and articular surface irregularity are optimally evaluated on CT which can also quantify tumour density. MRI demonstrates heterogeneous low signal matrix and is useful to assess decrease in size of cystic and/or soft tissue components of GCTB. A fat-suppressed fluid-sensitive MR sequence is important to detect tumour reactivation. Reduction in ^18^F-FDG-PET avidity represents an early sensitive sign of response to denosumab treatment. Regardless of imaging modality, close follow-up in a specialist centre and careful evaluation of nonresponders is necessary as local recurrence after cessation of denosumab treatment and malignant transformation of GCTB have been described.

## Introduction

Giant cell tumour of bone (GCTB) accounts for 5% of primary bone tumours and typically occurs in young adults (20–40 years old) after closure of the physis [1]. GCTB are commonly located in the distal radius, distal femur and proximal tibia, arising from the epi-metaphysis and extending up to the subchondral bone plate of the joint. In the axial skeleton, GCTB are most commonly found in the spine and sacrum [1].

The 2013 World Health Organization classification for bone tumours has a special category for osteoclastic giant cell-rich tumours, including benign lesions (of the small bones), intermediate and malignant GCTB [2].

These tumours contain three different cell types: a minority of mononuclear stromal cells (often pointed out as the true neoplastic component), reactive rounded mononuclear cells (of monocyte lineage) representing precursors of giant cells and a large number of multinucleated giant cells [3]. The mononuclear stromal cells express a receptor activator of nuclear factor kappa-B ligand (RANKL). The multinucleated osteoclastic giant cells express receptor activator of nuclear factor kappa-B (RANK). The RANKL stromal cells interact with RANK osteoclastic giant cells to induce osteoclast formation [4, 5]. The numerous osteoclastic giant cells in GCTB are responsible for extensive osteolysis seen in GCTB growth.

Medical treatment for GCTB includes anti-osteoclastic drugs such as zoledronic acid and other bisphosphonates which bind to bone mineral and act primarily by inhibiting osteoclast formation, migration and osteoclastic mediated bone resorption [6, 7]. Bisphosphonates have also been shown to promote apoptosis of both the GCTB osteoclastic giant cells and mononuclear stromal cells [8].

Denosumab is a RANK ligand inhibitor, a human monoclonal antibody that targets and binds with high affinity and specificity to RANKL. Denosumab specifically inhibits osteolysis by preventing RANKL-mediated formation and activation of multinucleated osteoclasts or giant cells from RANK-positive mononuclear preosteoclasts and macrophages [3]. Histological changes after treatment include a decrease in tumour giant cells of 90% or more, a marked reduction in RANKL-positive tumour stromal cells and increased fibro-osseous tissue formation [3]. The latter has been attributed to an indirect effect of denosumab on the osteoblasts, which alters the micro-environment of the tumour from osteolytic to bone forming [3].

GCTB may be locally aggressive, with local recurrence forming a challenging issue. The reported local recurrence rate varies widely and lies between 10 and 50% for intralesional resection (curettage) and 5% for wide resection [9–13]. Treatment with denosumab becomes an option in histologically confirmed GCTB where surgical resection may result in severe morbidity (such as joint reconstruction or amputation), or in patients who have an unresectable tumour (such as in the spine or sacrum) [3, 10, 12]. Short-term (3 months) pre-operative denosumab has also been reported to induce a neocortex and thereby facilitate surgical curettage [4]. Neoadjuvant denosumab therapy for a longer time period (median duration of 14 months) has been shown to downstage surgery to a less morbid surgical procedure [14]. Denosumab treatment of GCTB has been shown to limit tumour progression, reduce tumour size, reform mineralized bone, increase bone mineral density, reduce pain and analgesic use and improve functional status [9, 10, 15, 16]. Some authors continue denosumab treatment for 6 months after surgical curettage or en bloc resection of GCTB [14, 16]; however, there is currently no data clarifying whether maintenance denosumab therapy in the post-operative setting reduces the tumour recurrence rate.

The aim of this review is to illustrate the radiological findings of denosumab treatment on histologically confirmed GCTB. Imaging features on plain radiographs, computed tomography (CT), magnetic resonance imaging (MRI) and positron emission tomography-CT (PET-CT) will be discussed. Recognizing imaging features indicative of a positive tumoural response to denosumab in various tumour locations and on different imaging modalities is important for therapeutic decision-making [11]. There are currently no guidelines for optimal duration of denosumab treatment, and imaging findings with respect to the time on treatment are discussed. Conditions that interact with denosumab therapy such as hormonal upregulation during pregnancy, tumour recurrence and malignant transformation are reviewed.

## Plain radiographs

The Campanacci classification is a grading system for GCTB on plain radiographs [17]. Grade 1 lesions are latent and have a well-defined margin and an intact cortex. Grade 2 are active lesions with a relatively well-defined margin but no radiopaque rim. The cortex is thinned and moderately expanded. In grade 3 lesions, there are aggressive features such as indistinct borders and cortical destruction [18]. The Campanacci grading is useful for surgical planning but should not be interpreted as a prognostic factor, as there is no correlation between the Campanacci grade of the tumour and risk of local recurrence or metastases [13, 18, 19].

**Fig. 1:**
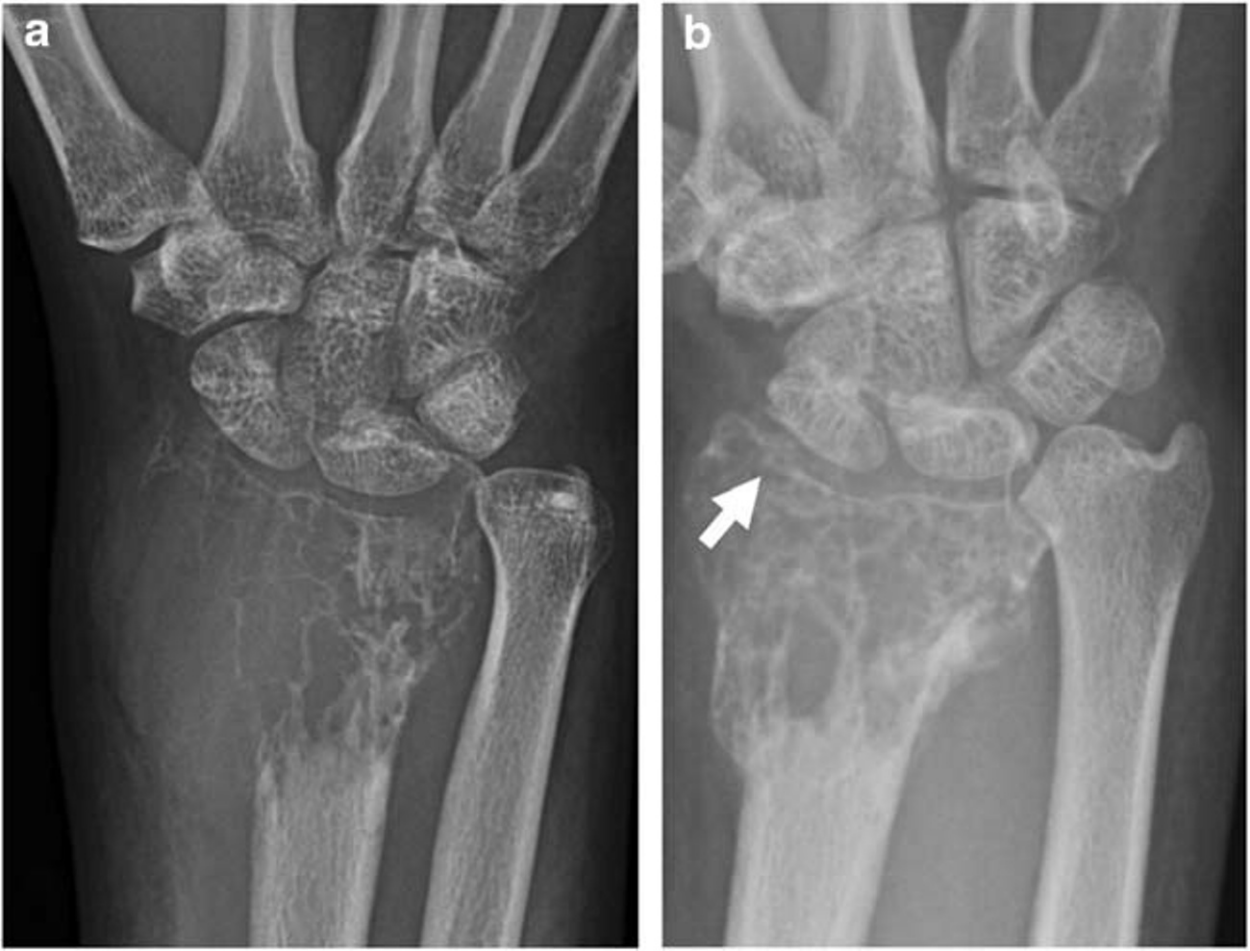
A 38-year-old female presented with wrist pain and swelling. Plain radiographs at presentation (**a**) and after 4 weeks on denosumab (**b**). **a** At presentation, a Campanacci grade 3 expansile lytic GCTB was found in the distal radius meta-epiphysis, extending up to the subchondral bone plate with destruction of the lateral radial cortex and radial styloid articular surface. **b** After 4 weeks of denosumab treatment, the cortex has reconstituted laterally with remodelling and irregularity of the distal radial articular surface (arrow). The soft tissue component of the lesion has markedly decreased in size. The distal radius shows an increase in matrix osteosclerosis

**Fig. 2:**
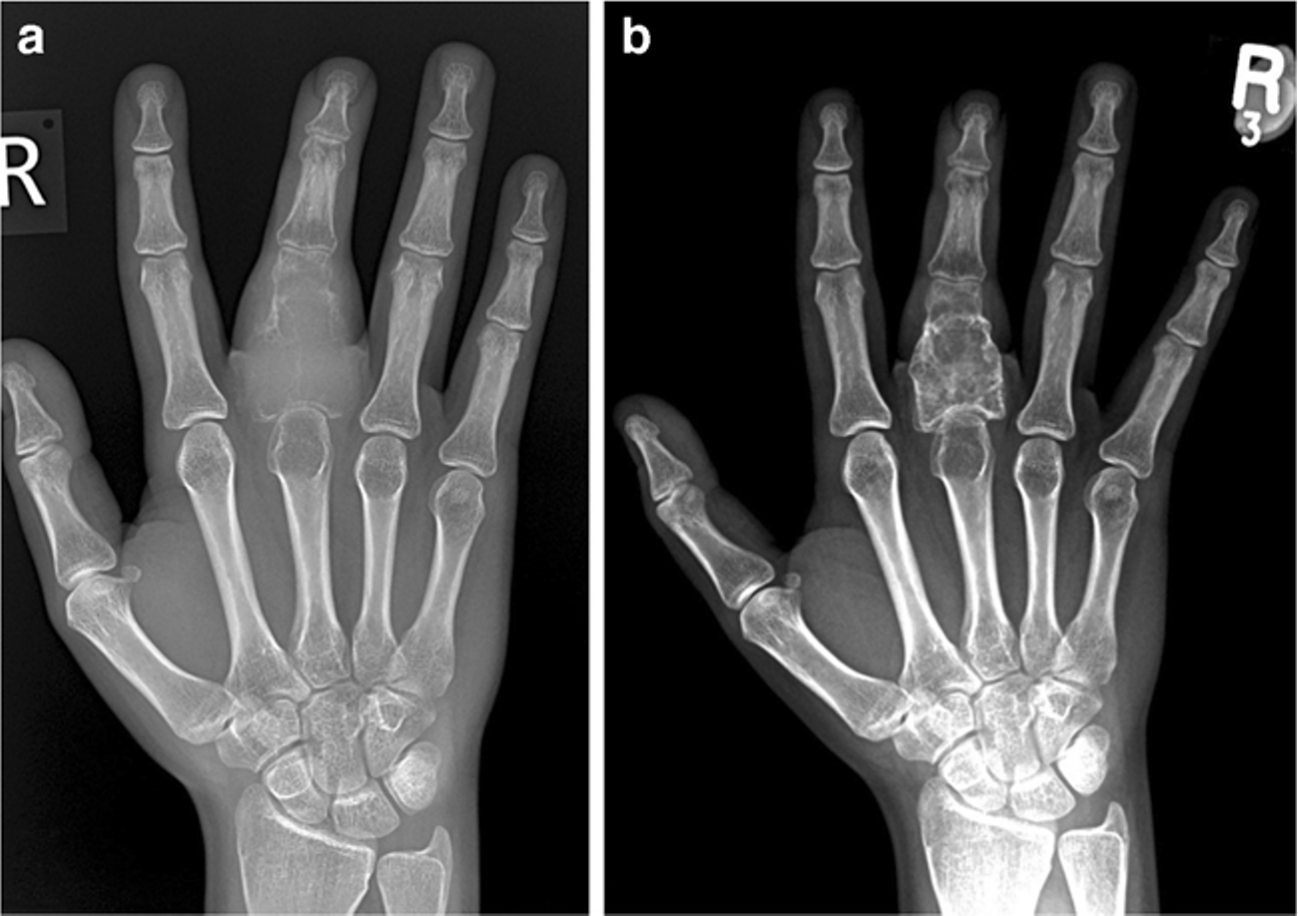
A 17-year-old girl presented with swelling of the middle finger. Plain radiographs at presentation (**a**) and after 12 weeks on denosumab (**b**). **a** At initial presentation, an expansile lytic GCTB is present in the middle finger proximal phalanx with cortical destruction. **b** After 12 weeks on treatment, the lesion has decreased in size with marginal neocortex formation and matrix sclerosis

**Fig. 3:**
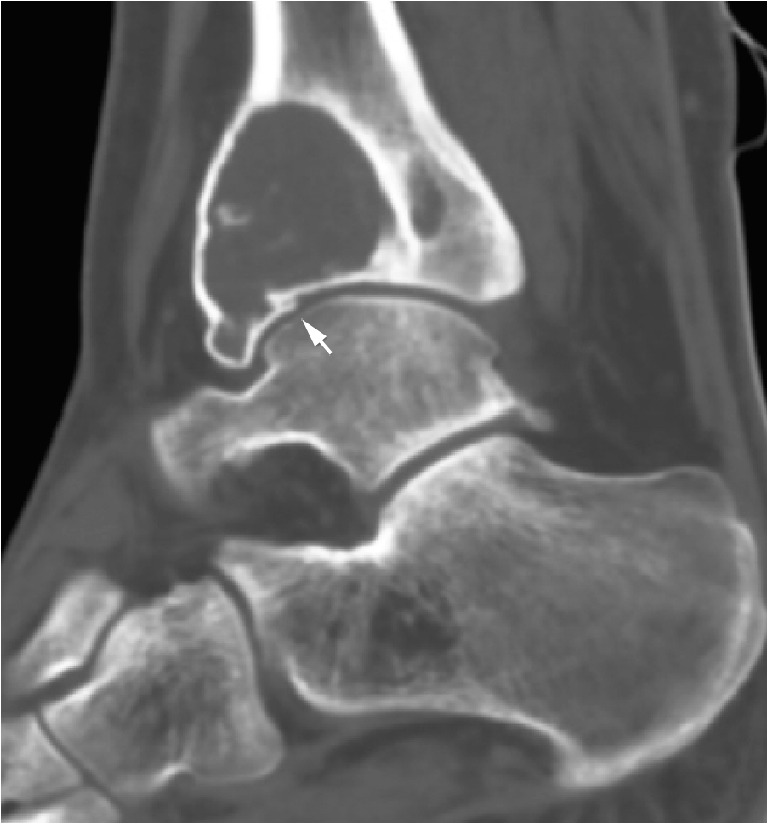
A 20-year-old male with known GCTB of the distal tibial epiphysis. CT sagittal reformat after 8 weeks on denosumab treatment. The GCTB is seen as a mildly expansile, eccentric lytic lesion with the development of marginal sclerosis and irregularity of the tibial articular surface anteriorly (arrow) while on denosumab

A key feature and aimed effect of denosumab treatment on plain radiographs is osteosclerosis seen as increased radiopacity within the area of tumour osteolysis [20]. This is often most pronounced along the periphery of the lesion, with well-defined marginal sclerosis and neocortex formation indicative of a positive response to denosumab [20]. Peripheral sclerosis is well described in the literature [3, 4, 11, 20–22] with treatment duration ranging from 7 weeks [11] to 2 years [22]. This marginal neocortex formation contributes to reconstitution of cortical and subarticular bone, which may effectively convert the lesion to a lesser Campanacci grade and facilitate intralesional curettage (Figs. 1 and 2) [4, 14, 20, 23]. The peripheral rim of mineralization may be thick and irregular, and remodelling of the articular surface can occur after denosumab treatment (Figs. 1 and 3) [4]. Articular surface irregularity potentially may give rise to early osteoarthritis, although there is no long-term data on this outcome as yet.

Intralesional osteosclerosis has been documented on plain radiographs as early as 2 weeks [11] after commencing denosumab, with varying degrees of internal matrix consolidation seen on plain radiographs increasing with treatment duration [4, 11, 14, 21, 22].

The incidence of pathological fracture is up to 30% in patients with GCTB; data to date does not indicate an increased fracture rate with denosumab treatment [9]. The effects of denosumab on pathological fractures in GCTB have not been well documented; however, initial reports describe complete healing of pathological fractures with pre-operative neoadjuvant denosumab treatment and the expected formation of peripheral sclerosis which may facilitate surgical excision [24, 25]. Intra-articular ectopic ossification has been described with an intra-articular pathological fracture within 1 month of commencing denosumab treatment. The intra-articular ossification demonstrated growth, in parallel with the radiographic tumour response to denosumab treatment, and histologically exhibited fibro-osseous components similar to those seen in the bone tumour. It is speculated that GCTB cells may seed into the joint in the presence of an intra-articular fracture and ossify in response to denosumab treatment [26].

## Computed tomography

**Fig. 4:**
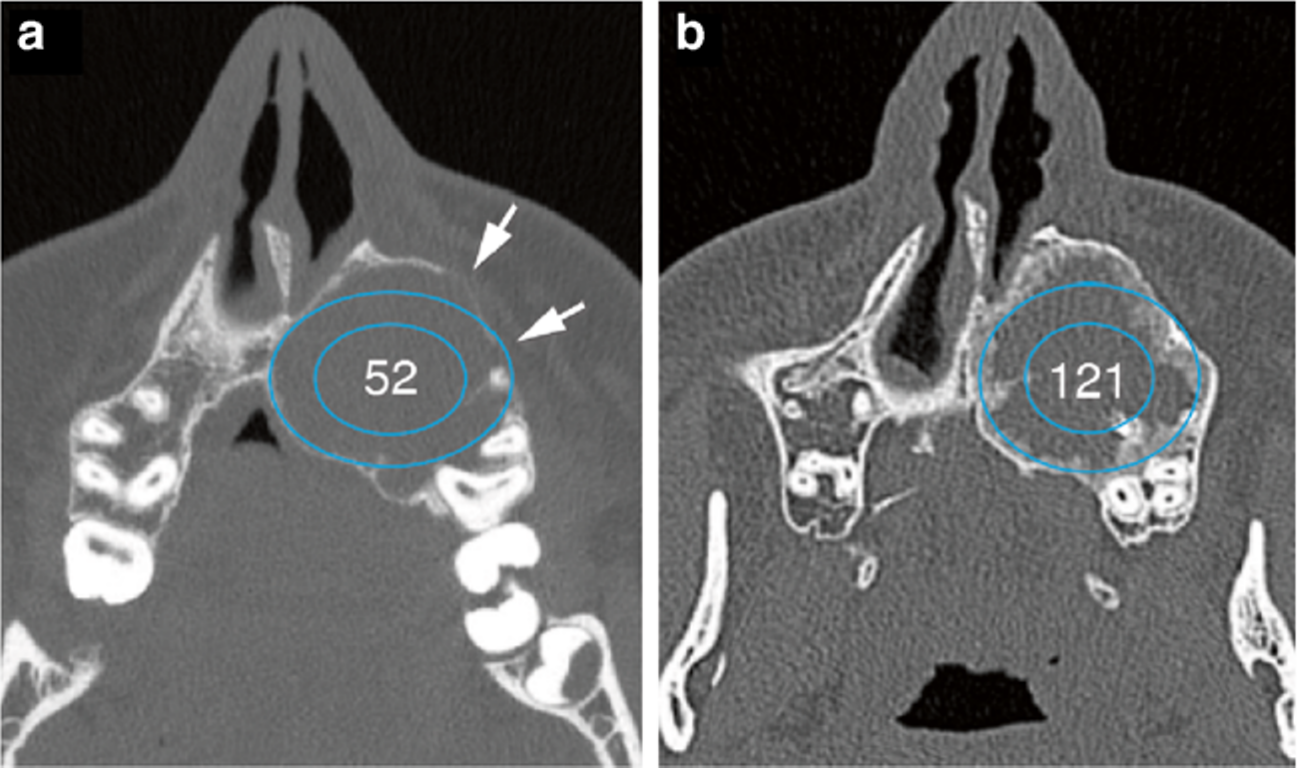
A 15-year-old male presented with swelling of the hard palate, which was first noticed by the dentist. Axial CT of the maxilla at presentation (**a**) and after 14 weeks on denosumab (**b**). **a** CT at presentation shows an expansile lytic GCTB between the roots of the teeth. The cortex was thinned with focal areas of cortical destruction (arrows). No ground glass or chondroid matrix was present in the lesion. The density of the tumour was 52 HU. **b** Follow-up CT after 14 weeks on treatment shows peripheral sclerosis surrounding the lesion with no cortical defects. The density of the tumour increased to 121 HU. Note that the angulation of the CTs varies slightly

As on plain radiographs, CT demonstrates tumour matrix and marginal sclerosis. Neocortex formation with reconstitution of areas of cortical destruction and subarticular bone is particularly well demonstrated with CT, which may be valuable for surgical planning (Figs. 4 and 12b).

CT adds the possibility to quantify the density of the tumour in Hounsfield units (HU), as a marker for the degree of sclerosis, indicative of response during denosumab treatment [27]. Inverse Choi density/size criteria have been shown to be more reliable than RECIST (i.e. maximum tumour size only) in assessing treatment response [9, 28].

We illustrate a GCTB located within the maxilla, where the HU increased from 52 to 121 (133% increase) after 14 weeks of treatment (Fig. 4).

In a case series including 8 patients, the average HU within a region of interest (ROI) in the GCTB increased from 45 to 110 HU (144% increase) after a mean of 5 (range 4–7) denosumab injections. ROIs were drawn semi-automatically (and checked manually) by tracing the outer edge of the tumour [27].

Engellau et al. documented that 99/124 patients (80%) had a > 15% increase in density using HU as a response parameter, reflecting the desired outcome of denosumab therapy [9]. HU evaluation showed that the increase in tumour density within 6 months on denosumab was consistent and sustained and that the mean HU values rarely decreased once an increase was observed [9].

GCTB may decrease in size on denosumab treatment [14], although a purely size based evaluation (e.g. using Response Evaluation Criteria in Solid Tumours (RECIST)) has been reported to be potentially insensitive in assessing response to denosumab [9, 28]. As far as we know, to date, no uniform imaging assessment criteria have been approved to specifically assess response to denosumab treatment in GCTB. To officially fulfil RECIST criteria, lytic bone lesions or mixed lytic-blastic lesions need to have a soft tissue component which can be measured on CT or MRI [29]. However, several clinical studies using RECIST criteria for CGTB did not specify in detail which part of the tumour they measured (i.e. only the soft tissue component or including the intra-osseous component) [9, 28].

Evaluation of tumour response to denosumab in two clinical studies using RECIST 1.1 did not adequately describe the effects of denosumab therapy on GCTB [9, 28], as these criteria found only a 25 to 35% response compared to a combination of reduction in ^18^F-FDG-PET avidity (82 to 96% response) and size/density measurements (71 to 76% response) [9, 28]. Thick peripheral neocortex formation, usually interpreted as a positive response to denosumab, may even increase tumour size, further substantiating that size alone is not a reliable measure to assess treatment response [5, 7, 8].

**Fig. 5:**
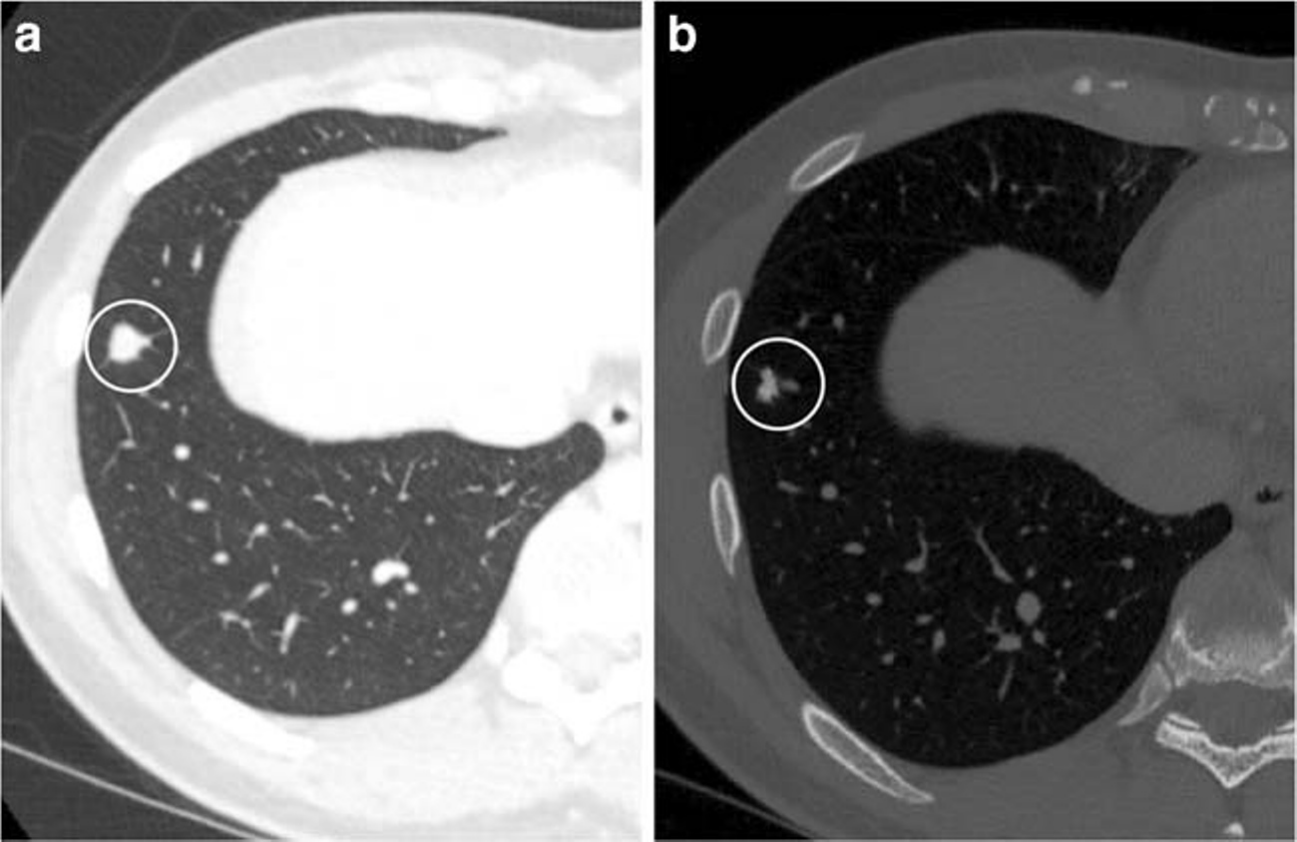
A 21-year-old male presented with a pathological fracture of the right hip secondary to a GCTB of the proximal femoral epiphysis. He developed histologically confirmed pulmonary metastases 3 months after diagnosis. CT of the chest at baseline (**a**) and after 6 months on denosumab (**b**). **a** Axial CT (lung window) of the chest at baseline demonstrates a right lower lobe pulmonary metastasis (circle). **b** Axial CT (bone window) of the chest performed 6 months after starting denosumab shows a decrease in size and ossification of the right lower lobe metastasis (within the circle)

Another important use of CT imaging is the detection and follow-up of pulmonary metastases that may occur in 1 to 6% of patients with GCTB [20]. Pulmonary metastases have been shown to respond to denosumab treatment with a decrease in size of the metastases [30]. Ossification of GCTB pulmonary metastases is also suggestive of response to denosumab (Fig. 5). Our findings are supported by Palmerini et al. who describe ossification of lung metastases at 3 months post denosumab treatment [31] and by a case report in which histopathology of a GCTB pulmonary metastasis following 10 months of denosumab treatment revealed the presence of osteoid with no osteoclast-type giant cells which parallels the findings of treated bone lesions [32].

## Magnetic resonance imaging

MRI is the imaging modality of choice to evaluate a decrease in size of a soft tissue component of GCTB, which may be indicative of a positive treatment response to denosumab (Figs. 7 and 8). Oguro et al. showed that maximum tumour diameter, assessed on T2-weighted images, decreased by 15% on average after 19 months on treatment, which is scored as stable disease according to RECIST [33].

**Fig. 6:**
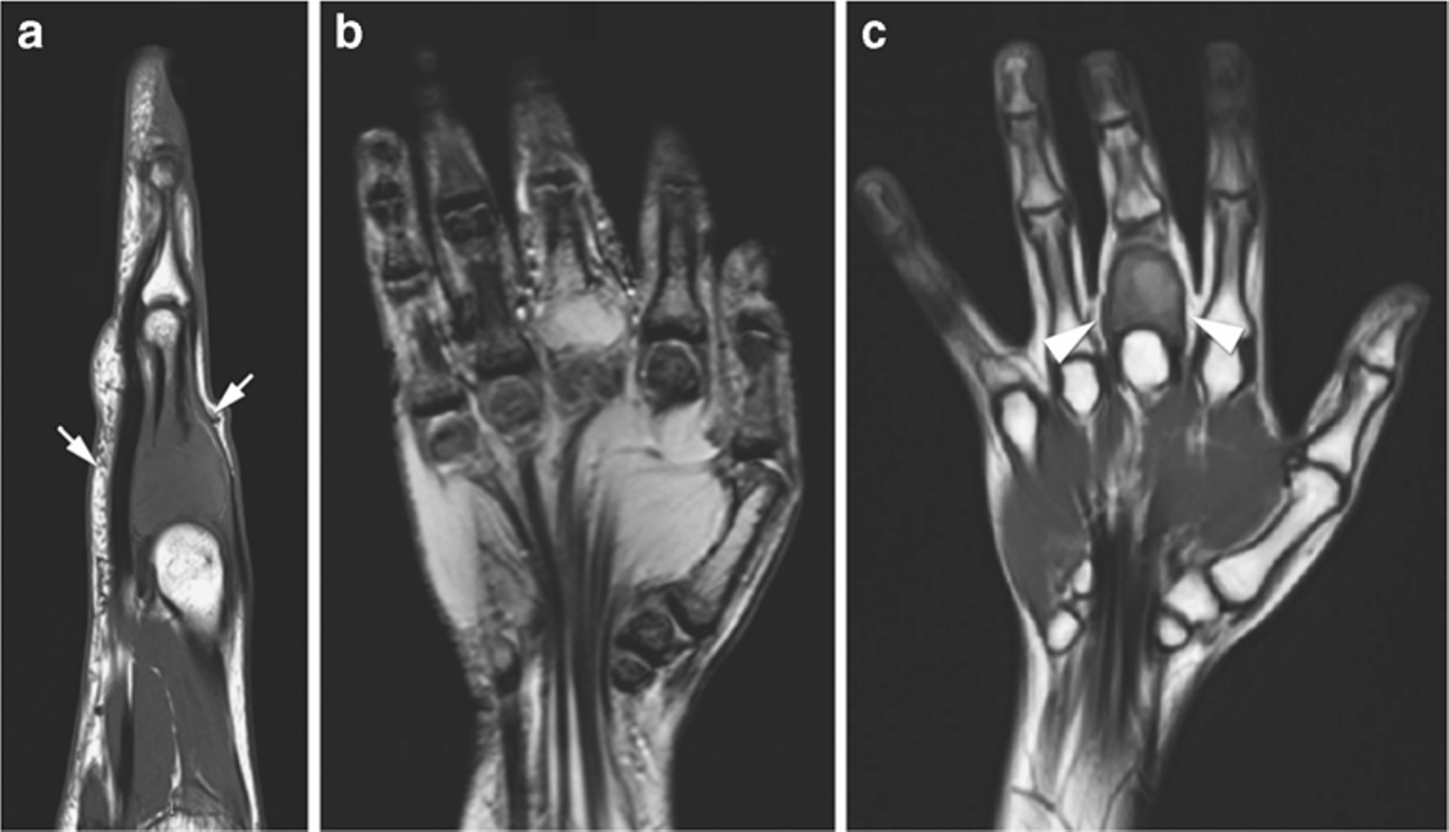
A 17-year-old girl presented with swelling of the middle finger (same patient as Fig. 2). MRI at presentation (**a**, **b**) and after 8 weeks on denosumab treatment (**c**). **a** Sagittal T1-weighted and **b** coronal proton density fat-saturated (PD-FS) MR images at presentation demonstrate an expansile, iso-intense T1 and high signal PD-FS GCTB in the proximal phalanx with cortical destruction and soft tissue extension (white arrows). **c** Coronal T1-weighted MR image after 8 weeks on denosumab shows decreased tumour size and overall lower signal intensity with a low signal intensity sclerotic rim (white arrowheads)

**Fig. 7:**
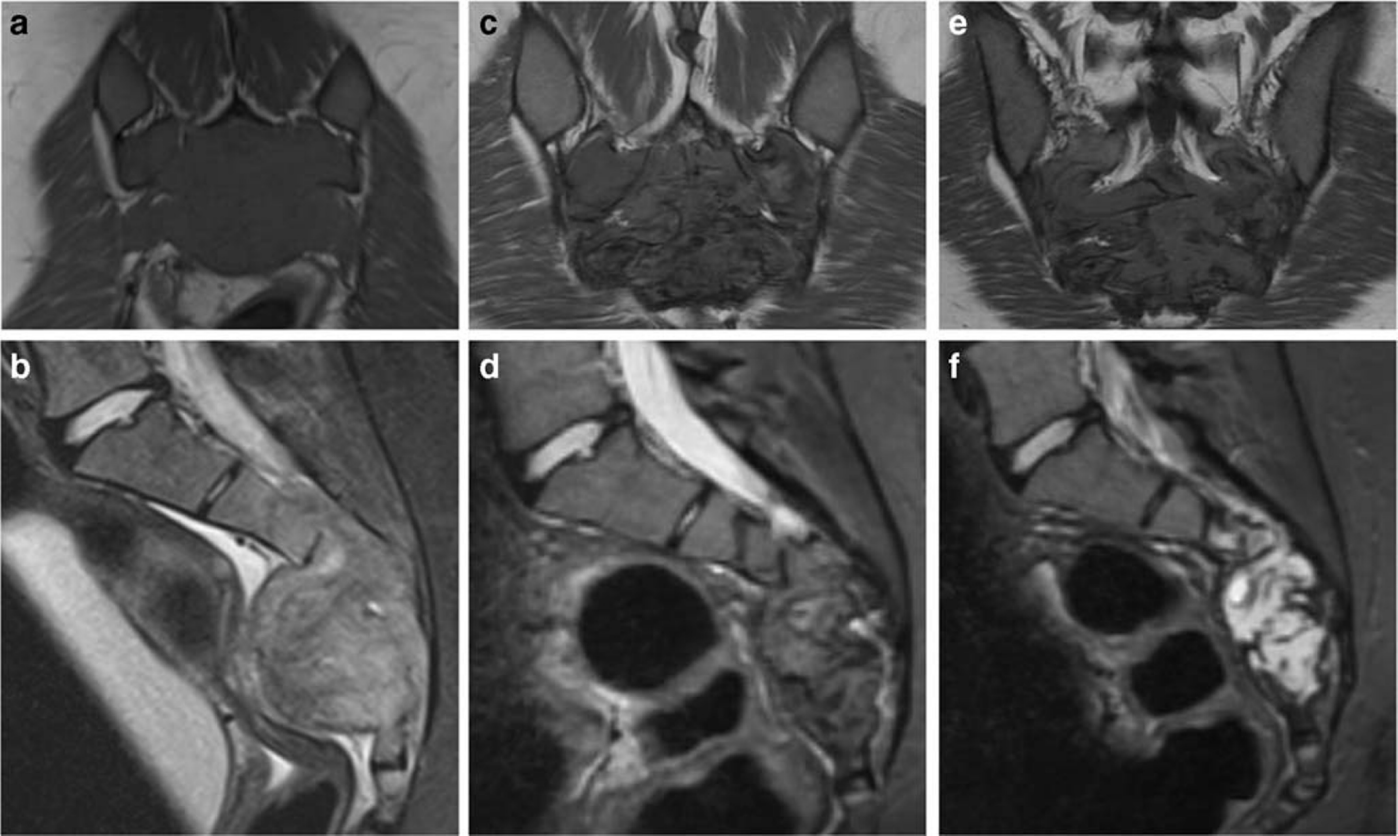
A 17-year-old female presented with cauda equina symptoms. MRI at presentation (**a**, **b**), after 8 months on denosumab (**c**, **d**) and after 13 months on denosumab (**e**, **f**). **a** Coronal T1-weighted MR image at presentation illustrates a large sacral GCTB, which is iso-intense to muscle on T1. **b** Sagittal T1 turbo inversion recovery magnitude (TIRM) MR image at presentation illustrates heterogeneous, predominantly high TIRM signal intensity in the tumour with a soft tissue component anteriorly, epidural and sacral foraminal extension. **c** Coronal T1-weighted MR image after 8 months on denosumab shows decreased signal intensity in the tumour, corresponding to sclerosis. **d** Sagittal T1 TIRM MR image after 8 months on denosumab shows the tumour has decreased in signal intensity and in size, which is mainly visible in the reduction of the soft tissue component. **e** The patient reported increased sacral pain after 13 months on denosumab treatment, and an interval MRI was performed. Coronal T1-weighted MR image demonstrates that tumour size and T1 signal intensity remain more or less unchanged. **f** Sagittal short tau inversion recovery (STIR) MR image after 13 months on denosumab demonstrates a marked increase in signal intensity of the tumour on the fluid-sensitive sequence, indicative of tumour reactivation. The patient was subsequently found to be pregnant and denosumab therapy was interrupted. Re-biopsy performed 3 months postpartum revealed numerous osteoclastic giant cells supporting the diagnosis of GCTB reactivation. Denosumab therapy was subsequently recommenced with a good clinical response and stable disease on treatment (2 year follow-up to date)

MR signal intensity is another marker to assess treatment response in GCTB. Previous studies have described that T1 signal intensity does not change significantly on treatment [4, 21]. From our experience, both T1 and T2 signal intensity decrease on denosumab therapy with the formation of low signal marginal sclerosis and internal matrix low signal (Figs. 6 and 7). The reduced MR signal intensity likely reflects the histological response to denosumab treatment with replacement of both multinucleated giant cells and mononuclear cells by fibroblast like spindle cells, dense fibro-osseous tissue and/or woven bone [3, 33].

Decreased T2-weighted MR signal has previously been described [4, 11, 21, 33] with demonstrable signal change from 7 weeks [11] after commencing denosumab treatment.

T2-weighted signal intensity also becomes more heterogeneous post-treatment as new areas of low T2 signal develop, likely corresponding to areas of central necrosis and new bone formation/sclerosis within the tumour [3, 21].

MRI findings of increased intralesional heterogeneity, cortical thickening and irregularity following short-term (7 weeks) denosumab therapy have previously been described and misinterpreted as disease progression [11]. These MR features are not uncommonly seen with denosumab treatment and represent an appropriate response to treatment.

**Fig. 8:**
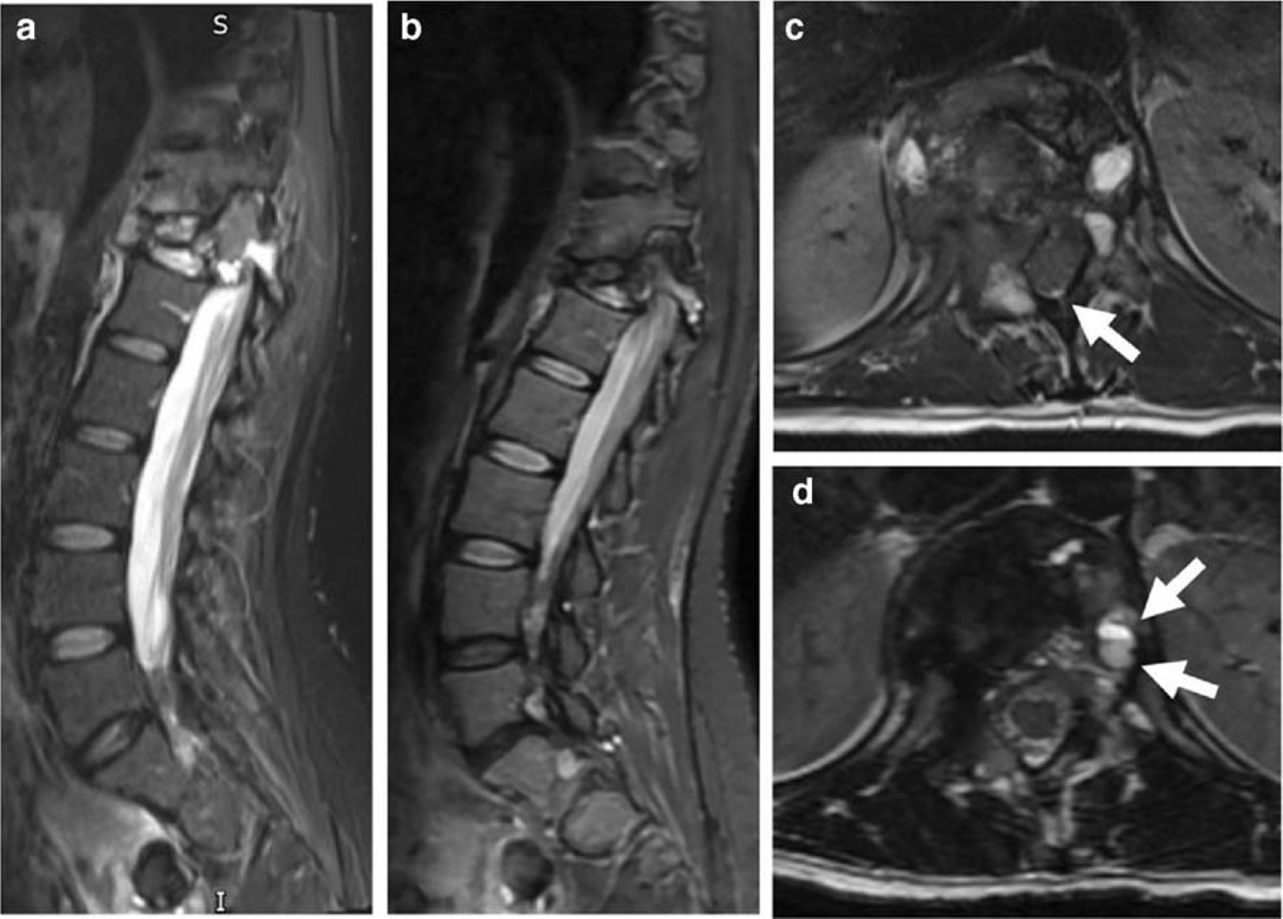
A 30-year-old female presented with back pain and a newly developed scoliosis. MRI at presentation (**a**, **c**) and after 8 weeks on denosumab (**b**, **d**). **a** Sagittal T2 TIRM MR image at baseline shows T12 vertebral collapse secondary to a GCTB with heterogeneous signal intensity and cystic components. **b** Sagittal T2 TIRM MR image after 8 weeks on denosumab demonstrates decreased size and signal intensity of the tumour, with further loss of height of the vertebral body. **c** Axial T2-weighted MR image at presentation shows tumour extension into the paravertebral soft tissues and epidural space. There is almost complete obliteration of the spinal canal with minimal remaining CSF posteriorly (white arrow). **d** Axial T2-weighted MR image after 8 weeks on denosumab shows decreased size and signal intensity of the tumour, with a more defined low signal margin and capacious spinal canal. One of the cystic components shows a fluid-fluid level, likely after haemorrhage (white arrows)

**Fig. 9:**
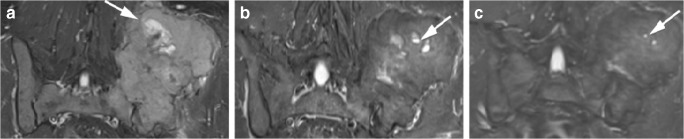
A 36-year-old man presented with an enlarging mass originating from the left ilium. MRI at presentation (**a**), after 12 weeks on denosumab (**b**) and after 20 weeks on denosumab treatment (**c**). **a** Coronal T1 TIRM MR image at baseline shows large cystic foci in the left iliac GCTB (arrow). **b** Coronal T1 TIRM MR image after 12 weeks on treatment demonstrates that the lesion appears smaller and of lower signal intensity, with decreased size of the cystic components (arrow). **c** Coronal T1 TIRM MR image after 20 weeks on treatment shows further reduction in size and signal intensity of the GCTB and the cysts are barely visible (arrow)

GCTB lesions may demonstrate central cystic areas, which are seen to decrease in size on treatment (Figs. 8 and 9); best appreciated on fluid-sensitive fat-suppressed sequences. Oguro et al. found that the cystic component of GCTB significantly decreased in size in 4/5 patients after treatment and is thought to be due to the blocking effect of denosumab on RANKL with resultant suppression of osteoclasts [33]. Fluid-fluid levels in keeping with secondary aneurysmal bone cyst formation have been described to be present in up to 14% of patients with GCTB [18, 20].

**Fig. 10:**
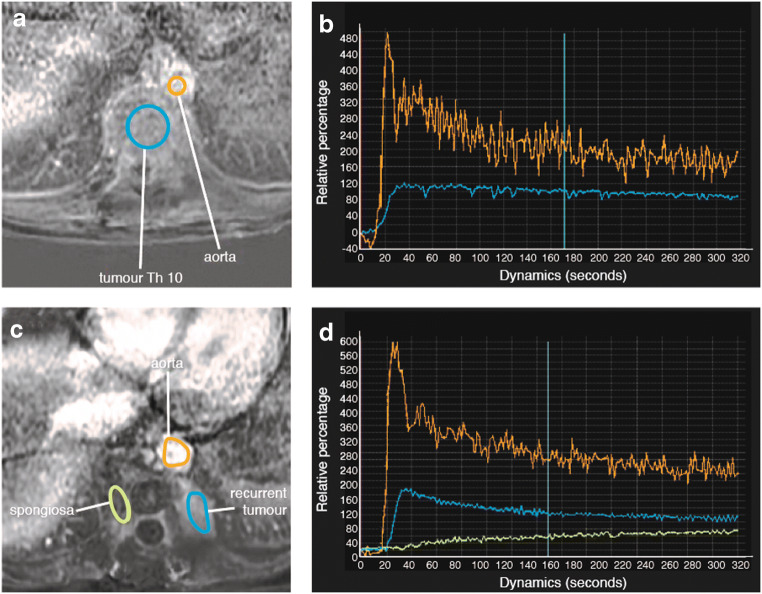
A 36-year-old female with GCTB of the T10 vertebra. Dynamic contrast-enhanced MRI at baseline (**a**, **b**) and follow-up MRI 1.5 years after intralesional resection (**c**, **d**). **a** Axial dynamic contrast-enhanced MR image at baseline demonstrates a region of interest (ROI) drawn in the tumour in Th10 (blue circle) and a ROI in the aorta to reference arterial enhancement (orange circle). **b** Time intensity curves (*X*-axis: time in seconds, *Y*-axis: relative percentage of enhancement) show the tumour (blue line) enhances almost simultaneously with the aorta (orange line) and it shows minor washout over 5 min. **c** Axial dynamic contrast-enhanced MR image 1.5 years after intralesional resection demonstrates a region of interest (ROI) drawn in a new left paravertebral soft tissue mass at Th10 (blue oval) and a ROI in the aorta to reference arterial enhancement (orange circle). There is a new right paravertebral soft tissue mass (green oval), in keeping with post-surgical spongiosa material as seen on CT (not shown). **d** Time intensity curves (*X*-axis: time in seconds, *Y*-axis: relative percentage of enhancement) show the new left paravertebral mass (blue line) enhances only 5 s after the aorta (orange line) and shows evident washout starting at *T* = 35 s, indicative of GCTB recurrence. This area was subsequently biopsied under CT guidance and recurrent GCTB was histologically confirmed. The green line represents the area of post-surgical spongiosa material and shows a small relative percentage of enhancement and slow enhancement over time

In our centre, intravenous gadolinium contrast is not routinely used for GCTB as from our experience contrast is not particularly helpful in assessment of response to denosumab. This is supported by Hakozaki et al. [21] and Oguro et al. [33], who found that the solid part of GCTB showed intense, comparable gadolinium enhancement both on pre- and post-denosumab scans. In accordance with our experience, Oguro et al. concluded that both shrinkage of the cystic component in the tumour and a decrease in T2 signal intensity (representing new bone formation) are more useful markers to assess treatment response [33]. Contrast enhancement and DWI/ADC were not found particularly useful; however, the study included only a small number of patients (*N* = 12) [33]. More research is needed to evaluate the value of contrast enhancement studies before and after denosumab treatment, as dynamic contrast enhancement (mean time intensity curves) may add more information [34]. Preliminary findings on dynamic contrast-enhanced MRI suggest that later enhancement followed by slower washout compared with the index MRI is indicative of response to treatment [23]. Dynamic contrast-enhanced MRI may also be useful to differentiate GCTB recurrence (fast enhancement and wash out curve) from post-surgical changes or bone graft material (slow and low relative percentage of enhancement over time) [35–37], as shown in Fig. 10.

Upregulation of GCTB while on denosumab therapy may occur in pregnancy due to hormonal influences causing a rapid increase in tumour growth and activity [38, 39]. This may be seen as increased signal on fat-suppressed fluid-sensitive MR sequences, whereas T1-weighted signal may remain static (Fig. 7). Two case reports of GCTB recurrence during pregnancy in the capitate and in the spine described rapid expansion of the tumour during pregnancy [38, 39]. Females on denosumab should be advised to avoid pregnancy and take appropriate contraceptive precautions as there is evidence that denosumab is associated with increased stillbirth and decreased body weight gain, growth and development in studies of animal infants exposed in utero [40, 41]. Skeletal effects observed in infant monkeys exposed in utero are consistent with the anticipated pharmacological activity of denosumab with inhibition of bone resorption and impaired bone remodelling during skeletal development resulting in an osteoclast-poor osteopetrotic like skeletal phenotype at birth and in the early postnatal period [42].

## PET-CT

**Fig. 11:**
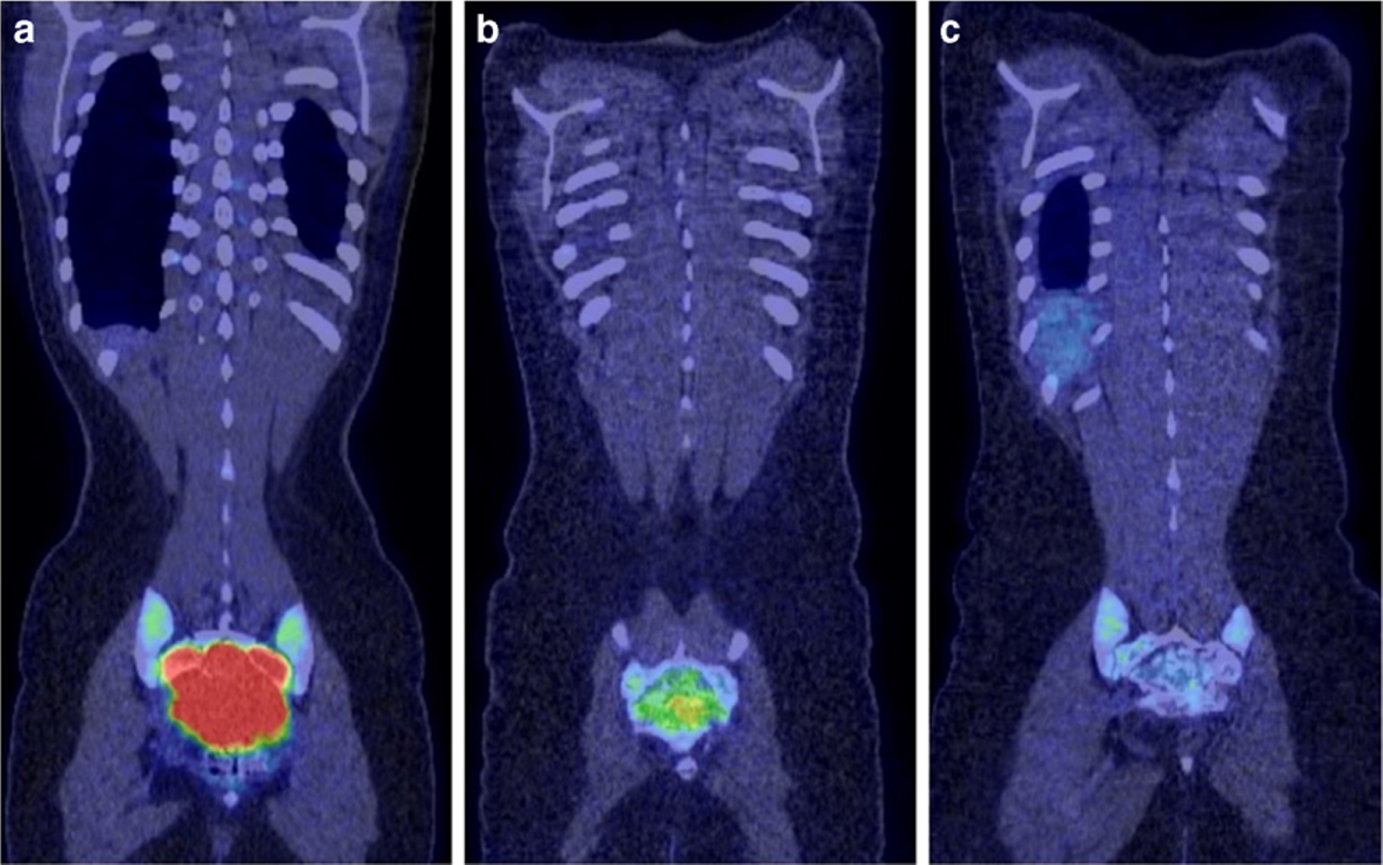
A 17-year-old female with a sacral GCTB (same patient as Fig. 7). PET-CT at baseline (**a**), after 8 weeks on denosumab (**b**) and after 16 weeks on denosumab (**c**). **a** Coronal fused PET-CT at baseline shows marked ^18^F-FDG uptake in the sacral GCTB which had an SUV^max^ of 16.3. No metastases were present. **b** Coronal fused PET-CT after 8 weeks on treatment demonstrates that the SUV^max^ had decreased to 4.4. **c** Coronal fused PET-CT after 16 weeks on denosumab demonstrates a complete response

**Fig. 12:**
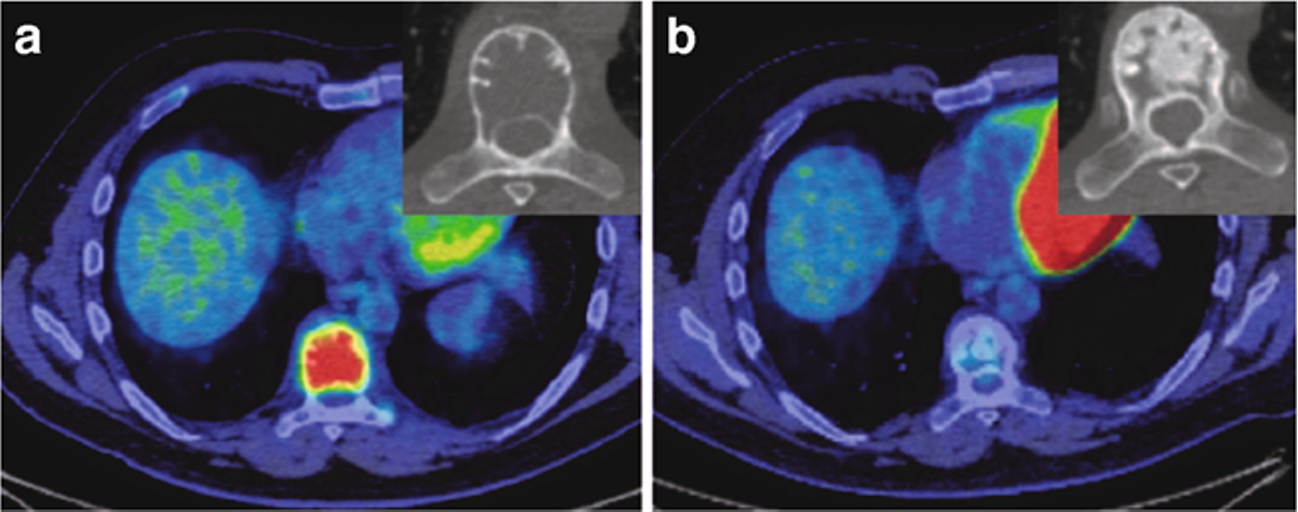
A 42-year-old man with a GCTB of the T8 vertebra. Fused axial PET-CT image and a detail from the axial CT (bone window) are shown at baseline (**a**) and after 16 weeks on denosumab (**b**). **a** Fused PET-CT at baseline demonstrates marked ^18^F-FDG uptake in a lytic T12 GCTB with an SUV^max^ of 9. **b** Fused PET-CT after 16 weeks on treatment shows no increased ^18^F-FDG uptake in the GCTB when compared with the adjacent vertebrae, indicative of a good response. The unfused axial CT image illustrates progressive peripheral sclerosis and increased matrix osteosclerosis in the tumour with treatment

PET-CT is a valuable addition for assessment of response to denosumab treatment, as not only the SUV^max^ pre- and post-treatment can be assessed on fused images but also the development of new bone formation can be monitored on the accompanying unfused CT [4, 10, 21]. In our centre, whole-body PET-CTs are performed at baseline, at 8 weeks and 16 weeks on denosumab treatment to assess response. Treatment response with a significant decrease in SUV^max^ can be detected as early as 8 weeks (Fig. 11). Unfused CT images demonstrate increasing marginal and matrix osteosclerosis in the tumour (Fig. 12).

Our findings are in agreement with the study by Engellau et al., in which reduction in ^18^F-FDG-PET avidity was shown to be an early and universal sign of response to denosumab treatment [9]. A reduction in ^18^F-FDG-PET avidity was found to predict a favourable tumour response and sustained tumour control with denosumab treatment, which did not vary with lesion location. Given the rarity of nonresponse to denosumab in GCTB, the authors suggest that in the case of no decrease or even an increase in SUV^max^ during denosumab treatment, an aggressive lesion such as a giant cell-rich osteosarcoma needs to be excluded histologically [9].

Using European Organisation for Research and Treatment of Cancer (EORTC) criteria, which incorporate changes in ^18^F-FDG uptake, Ueda et al. also demonstrated a good response to denosumab in the majority of patients, which was found to be compatible with clinical improvement [28].

In a case series of 5 patients with GCTB of the distal radius, PET-CT demonstrated a good response to short term (3 months) treatment with denosumab with the SUV^max^ decreasing from 14.8 pre-treatment to 4.7 at 2 months [3].

The high baseline ^18^F-FDG uptake in GCTB is most likely related to the high metabolic activity of tumour giant cells [10, 21]. Decrease in ^18^F-FDG uptake on denosumab treatment corresponds to a significant reduction in tumour giant cells with phase 2 studies demonstrating > 90% elimination of giant cells in all patients on histological evaluation after 3 and 7 months on denosumab treatment [3, 10].

GCTB metastases to the lungs can also be assessed and monitored with PET-CT: this modality offers the benefit of both morphological assessment (on the CT) and functional assessment (SUV^max^ on PET) with treatment.

## DEXA scan

Denosumab is an approved drug for the treatment of osteoporosis (as an alternative to bisphosphonates), although the dose for this indication is only 60 mg per 6 months. For the treatment of GCTB, the dose is 120 mg on days 1, 8, 15 and 29 followed by every 4 weeks. The assessment of bone mineral density (BMD) as a marker for treatment response in GCTB may be promising. In a case series including three patients, dual-energy X-ray absorptiometry (DEXA) scans were used for evaluation of treatment effect with denosumab. By applying a region of interest (ROI) in the tumour on a DEXA scan, bone mineral density (BMD) was measured and compared at several time points [15]. All three patients had a steadily increasing BMD in the tumour throughout denosumab treatment. BMD in the lumbar spine and hip (used as control areas for standardized BMD measurement) did not increase like the ROIs in the GCTB, which may be explained by the patients’ normal baseline BMD. DEXA scans may be used to help decide on the optimum duration of treatment and the timing of surgery in future studies. Exact timing of DEXA scans after treatment needs to be established; however, in this study, one patient was shown to have a 15% increase in BMD only 9 weeks after the first injection with denosumab and a second patient showed a 7.5% increase in BMD after 7 weeks of treatment [15].

## Duration of denosumab treatment and imaging findings

In GCTB cases for whom surgical resection is the primary treatment of choice, denosumab may be given as a neoadjuvant chemotherapy to facilitate tumour resection [4, 11, 14, 23]. Pre-operative denosumab is given to stop tumour osteolysis, increase bone mineral density (to prevent pathological fractures), increase marginal sclerosis and reconstitute the articular surface to obtain a better tumour resection margin.

However, optimal treatment duration is a complex matter, as with increasing time on treatment marked peripheral new bone formation and intralesional sclerosis may make complete curettage and tumour removal more challenging [3]. In addition, residual sclerotic bone may contain neoplastic stromal cells that may become active and express RANKL after denosumab treatment is stopped, leading to local recurrence [23, 43].

Performing surgery 3–4 months after starting denosumab to prevent too thick a rim of bone from forming has been considered to make complete tumour removal more feasible [4, 23].

Studies to date have demonstrated intralesional consolidation on plain radiographs as early as 2 weeks [11], decrease in T2-weighted MR signal at 7 weeks [11], increase in BMD by 7.5% on DEXA at 7 weeks [15], increase in average HU by > 15% on CT at 8 weeks [27] and significant reduction in ^18^F-FDG avidity on PET-CT at 8 weeks [4, 9].

In studies by Ueda et al. (*N* = 17) and Engellau et al. (*N* = 190) using RECIST (maximum tumour diameter), EORTC (SUV^max^ on PET-CT) and inverse Choi criteria (tumour size and density on CT or MRI) to assess GCTB response to denosumab, median time to tumour response based on the best response using any scoring system was 3 months in both studies [9, 28]. This time period did not vary for PET-CT (EORTC) or CT/MRI (inverse Choi criteria) [9, 28].

A study by McCarthy et al. (*N* = 5) also indicates that GCTB of the distal forearm responds to 3 months of denosumab treatment with sufficient marginal sclerosis to facilitate surgical curettage in all cases [4].

Although studies suggest a response to denosumab in most cases by 3 months, in the context of neoadjuvant denosumab treatment aimed to downstage the tumour, treatment duration may depend on the planned type of surgical resection (e.g. longer treatment duration for en bloc resection than for intralesional curettage) and on the surgeon’s preference [23].

Ongoing research is needed to determine how long to continue treatment in inoperable cases, whether long-term definitive control can be achieved, and to assess long-term safety and side effects [16].

## Recurrent GCTB during or post denosumab treatment

Age at diagnosis has been shown to be an independent risk factor for GCTB recurrence, regardless of stage of disease and chosen surgical treatment. Patients under 25 years at diagnosis are the group with the greatest risk of local recurrence [13].

GCTB with soft tissue extension and tumours of the axial skeleton have also been shown to have a higher risk of local recurrence [23]. An example of recurrence in the axial skeleton is shown in Fig. 10.

Although denosumab-treated GCTB specimens show a decrease of > 90% in tumour giant cells and a reduction in neoplastic stromal cells, recurrence may in part be explained by persistence of a small volume of neoplastic stromal cells which continue to proliferate albeit at a slower rate than in untreated GCTB [3, 10, 23, 43]. Further affirmation of persistence of tumour cells was shown by Girolami et al., who found the same H3F3A-mutated mononuclear cell population in two cases before and after denosumab treatment [44].

Soft tissue recurrence usually arises adjacent to the primary tumour through the seeding of tumour cells at the time of surgery or due to a pathological fracture. Soft tissue recurrence can become apparent during denosumab treatment due to the development of a peripheral rim of ossification over time [45].

Although the ossification is described to emerge simultaneously with the start of denosumab therapy and gradually increase with treatment, it remains unclear whether the marginal mineralization is related to denosumab as this finding has also been reported in soft tissue recurrent GCTB without denosumab treatment [46–48]. In these cases, peripheral ossification varied in thickness from a thin shell to a thick rind surrounding the recurrent soft tissue mass. Without denosumab treatment, such ossification is pathologically considered to be the result of metaplastic bone formation; it has been suggested that neoplastic stromal cells and osteoclastic-like giant cells release transforming growth factor β1 and transforming growth factor β2 when exposed to an extraosseous environment, which stimulates osteoblastic differentiation and bone formation [49]. Local recurrence tends to occur within 7–9 months after stopping denosumab treatment [23]; however, aggressive local recurrence within 2 months after cessation of long-term denosumab therapy has been described [22]. A potential mechanism to explain rapid recurrence, despite an initial good response to denosumab, could be that by binding RANKL, denosumab treatment drives the osteoclast-like giant cells to overexpress RANK and the stromal cells to overexpress RANKL. Then, once treatment is stopped, any residual tumour cells go into overdrive, leading to rapid growth and loss of the previously formed osteosclerosis [22].

There are currently no established guidelines for optimal frequency and modality of radiological follow-up post denosumab treatment. In our centre, we perform baseline imaging within 3 months of discontinuing denosumab, followed by imaging at 6 and 12 months or sooner if new onset or worsening clinical symptoms. Our clinicians favour plain radiographs and MRI for extremity GCTB and MRI for axial lesions. Although follow-up protocols may differ in various sarcoma centres and practitioners may prefer a different combination of imaging modalities, close clinical and imaging follow-up is required, especially in the first year after stopping denosumab treatment.

Evaluation for GCTB recurrence after stopping denosumab treatment requires thorough inspection and careful comparison of follow-up imaging with the initial baseline post denosumab images. New areas of osteolysis, reflecting recurrent osteoclastic activity, is a key imaging finding of GCTB recurrence. Other radiological findings of recurrent GCTB include new areas of bone destruction, tumour growth with expansion of bone and a new soft tissue component [22]. These findings may be seen on interval follow-up imaging; however, new onset of clinical symptoms, particularly pain and swelling, is the most common presentation of recurrence [23].

## Malignant or sarcomatous transformation during denosumab treatment

Malignant transformation in GCTB can be categorized as primary (a sarcoma occurring concomitantly within the conventional GCTB) or secondary (high-grade tumours, occurring after radiotherapy or surgery) [50, 51].

In histologically typical GCTB, sarcomatous change has been reported as extremely rare, less than 1% [1, 20, 52]. The recently published largest prospective clinical trial to date (*n* = 532) reported 1% of confirmed sarcomatous transformation in GCTB patients on denosumab [16]. Of the 5 patients with sarcomatous transformation, 1 had secondary malignant transformation (post radiation sarcoma).

Several cases of sarcomatous transformation in *recurrent* GCTB have been described, for example in the tibia with transformation into a high-grade pleomorphic sarcoma after 13 months on denosumab treatment [52] and transformation into a high-grade osteosarcoma in the ischium after 6 months on treatment [53]. Transformation into different types of sarcoma has been described, including undifferentiated pleomorphic sarcoma, fibrosarcoma and osteosarcoma [12, 54].

The key clinical findings that point towards sarcomatous transformation are worsening or new onset of pain and growth of the lesion during treatment. Absence of expected radiological findings during denosumab treatment, particularly lack of peripheral or central matrix osteosclerosis formation and decreased ^18^F-FDG-PET avidity, should alert clinicians to the possibility of misdiagnosis or sarcomatous change. Secondary malignant GCTB may present as an aggressive osteolytic tumour with cortical destruction and a soft tissue component [55]. Unfortunately, imaging findings for malignant transformation are not specific, given that both benign and malignant GCTB can show aggressive features [50, 51, 54].

It has been shown that sarcomatous transformation occurs after a shorter time interval (mean of 1 year) when related to denosumab treatment than when it is radiotherapy related, in which case the interval is longer (mean of 8 years) [53]. This data confirms the need for close clinical and imaging follow-up especially during the first year of treatment with denosumab.

However, whether sarcomatous transformation in recurrent GCTB is a causal or coincidental phenomenon is unclear. For example, it may be that the patients with recurrence represent a group with worse prognosis and a higher baseline risk of sarcomatous transformation. Unfortunately, as yet no biological hypothesis exists that explains the association between denosumab treatment and malignant transformation.

Misdiagnosis of the primary or of the recurrent tumour is another pitfall [16]. Histopathology of denosumab-treated GCTB tumours may resemble that of low-grade central osteosarcoma due to new bone formation in a fibrous background [56]. Benign multinucleated giant cells may be present in up to 36% of low-grade central osteosarcomas, making the distinction more challenging [57]. Genetic analyses such as the H3F3A mutation (present in giant cell-rich sarcomas) may be needed to distinguish the two tumours. These findings underline the need of a specialized tertiary sarcoma referral centre so that cases can be discussed and reviewed with expert bone tumour pathologists. It is essential that the correct clinical context of biopsy specimens is provided to the pathologist, specifically details regarding radiotherapy, denosumab therapy and clinical and radiological response to treatment.

## Conclusions

Tumour size by itself is not a good marker for response to denosumab treatment. The development of a peripheral sclerotic rim of neocortex and varying degrees of matrix osteosclerosis are indicative of a positive response to denosumab treatment. This may be well seen on plain radiographs for GCTB of the extremities and on CT for axial lesions.

Reconstitution of cortical and subarticular bone, articular surface remodelling and irregularity are optimally evaluated on CT which may be valuable for surgical planning. CT studies can also add density measurement (HU) to potentially quantify the degree of new bone formation. Decreased size and ossification of pulmonary metastases on CT can be interpreted as treatment response to denosumab.

Positive treatment response on MRI may be seen as a low signal sclerotic margin, increased T2 heterogeneity and matrix low signal on all sequences. MRI is particularly useful to evaluate decrease in size of cystic and/or soft tissue components of GCTB indicative of a positive tumour response to denosumab. Increased T2 MR signal while on treatment may indicate tumour reactivation. A fat-suppressed fluid-sensitive sequence in follow-up MRI protocols is useful to detect tumour reactivation (such as in pregnancy) and decreased cystic components which may not be apparent on T1-weighted sequences.

Reduction in ^18^F-FDG-PET avidity represents an early and sensitive sign of response to denosumab treatment.

Clinical trials using specified radiological parameters suggest the median time to objective tumour response is 3 months. Further studies are required to determine the optimal imaging technique and frequency of radiological follow-up, as currently no guidelines exist. Regardless of imaging modality used, close clinical follow-up and careful radiological evaluation of nonresponders is necessary. New onset or worsening clinical symptoms, particularly pain and swelling; tumour growth with new areas of osteolysis; absence of expected radiological findings on denosumab treatment, particularly lack of peripheral and central matrix osteosclerosis; and no decrease in ^18^F-FDG-PET avidity should alert the radiologist to the possibility of tumour progression, aggressive clinical variant, sarcomatous change or misdiagnosis which needs to be excluded histologically. Therefore, GCTB requiring denosumab treatment should be followed up at expert centres within a multidisciplinary team.

## References

[1] Verschoor AJ, Bovee JVMG, Mastboom MJL, Sander Dijkstra PD, Van De Sande MAJ, Gelderblom H (2018). Incidence and demographics of giant cell tumor of bone in The Netherlands: first nationwide Pathology Registry Study. Acta Orthop England.

[2] CDM F, Bridge JA, MF HPCW (2013). WHO classification of tumours of soft tissue and bone.

[3] Branstetter DG, Nelson SD, Manivel JC, Blay J-Y, Chawla S, Thomas DM (2012). Denosumab induces tumor reduction and bone formation in patients with giant-cell tumor of bone. Clin Cancer Res United States.

[4] McCarthy CL, Gibbons CLMH, Bradley KM, Hassan AB, Giele H, Athanasou NA (2017). Giant cell tumour of the distal radius/ulna: response to pre-operative treatment with short-term denosumab. Clin Sarcoma Res England.

[5] Kitazawa R, Haraguchi R, Fukushima M, Kitazawa S (2018). Pathologic conditions of hard tissue: role of osteoclasts in osteolytic lesion. Histochem Cell Biol Germany.

[6] Lipplaa A, Kroep JR, van der Heijden L, Jutte PC, Hogendoorn PCW, Dijkstra S (2019). Adjuvant zoledronic acid in high-risk giant cell tumor of bone: a multicenter randomized phase II trial. Oncologist United States.

[7] Balke M, Campanacci L, Gebert C, Picci P, Gibbons M, Taylor R (2010). Bisphosphonate treatment of aggressive primary, recurrent and metastatic Giant Cell Tumour of Bone. BMC Cancer England.

[8] Cheng YY, Huang L, Lee KM, Xu JK, Zheng MH, Kumta SM (2004). Bisphosphonates induce apoptosis of stromal tumor cells in giant cell tumor of bone. Calcif Tissue Int United States.

[9] Engellau J, Seeger L, Grimer R, Henshaw R, Gelderblom H, Choy E (2018). Assessment of denosumab treatment effects and imaging response in patients with giant cell tumor of bone. World J Surg Oncol England.

[10] Thomas D, Henshaw R, Skubitz K, Chawla S, Staddon A, Blay J-Y (2010). Denosumab in patients with giant-cell tumour of bone: an open-label, phase 2 study. Lancet Oncol England.

[11] von Borstel D, Taguibao RA, Strle NA, Burns JE (2017). Giant cell tumor of the bone: aggressive case initially treated with denosumab and intralesional surgery. Skelet Radiol Germany.

[12] Xu SF, Adams B, Yu XC, Xu M (2013). Denosumab and giant cell tumour of bone—a review and future management considerations. Curr Oncol Canada.

[13] Klenke FM, Wenger DE, Inwards CY, Rose PS, Sim FH (2011). Giant cell tumor of bone: risk factors for recurrence. Clin Orthop Relat Res United States.

[14] Rutkowski P, Ferrari S, Grimer RJ, Stalley PD, Dijkstra SPD, Pienkowski A (2015). Surgical downstaging in an open-label phase II trial of denosumab in patients with giant cell tumor of bone. Ann Surg Oncol United States.

[15] Veng C, Jørgensen PH, Krog-Mikkelsen I, Stilling M. Measurement of bone mineral density as an efficacy marker in denosumab treatment of giant cell tumour of bone. BMJ Case Rep. 2017;2017:bcr2017220369. 10.1136/bcr-2017-220369 PMC5665203 29066635

[16] Chawla S, Blay J-Y, Rutkowski P, Le Cesne A, Reichardt P, Gelderblom H (2019). Denosumab in patients with giant-cell tumour of bone: a multicentre, open-label, phase 2 study. Lancet Oncol England.

[17] Campanacci M, Baldini N, Boriani S, Sudanese A (1987). Giant-cell tumor of bone. J Bone Joint Surg Am United States.

[18] Mavrogenis AF, Igoumenou VG, Megaloikonomos PD, Panagopoulos GN, Papagelopoulos PJ, Soucacos PN (2017). Giant cell tumor of bone revisited. SICOT-J France.

[19] Takeuchi A, Tsuchiya H, Niu X, Ueda T, Jeon D-G, Wang EHM (2011). The prognostic factors of recurrent GCT: a cooperative study by the Eastern Asian Musculoskeletal Oncology Group. J Orthop Sci Japan.

[20] Chakarun CJ, Forrester DM, Gottsegen CJ, Patel DB, White EA, Matcuk GRJ (2013). Giant cell tumor of bone: review, mimics, and new developments in treatment. Radiographics United States.

[21] Hakozaki M, Tajino T, Yamada H, Hasegawa O, Tasaki K, Watanabe K (2014). Radiological and pathological characteristics of giant cell tumor of bone treated with denosumab. Diagn Pathol England.

[22] Matcuk GRJ, Patel DB, Schein AJ, White EA, Menendez LR (2015). Giant cell tumor: rapid recurrence after cessation of long-term denosumab therapy. Skelet Radiol Germany.

[23] Gaston CL, Grimer RJ, Parry M, Stacchiotti S, Dei Tos AP, Gelderblom H, et al. Current status and unanswered questions on the use of Denosumab in giant cell tumor of bone. Clin Sarcoma Res [Internet] England. 2016;6:15. Available from: http://clinicalsarcomaresearch.biomedcentral.com/articles/10.1186/s13569-016-0056-0. 10.1186/s13569-016-0056-0 PMC5022265 27651889

[24] Medellin MR, Fujiwara T, Tillman RM, Jeys LM, Gregory J, Stevenson JD (2018). Prognostic factors for local recurrence in extremity-located giant cell tumours of bone with pathological fracture. Bone Joint J England.

[25] Traub F, Singh J, Dickson BC, Leung S, Mohankumar R, Blackstein ME (2016). Efficacy of denosumab in joint preservation for patients with giant cell tumour of the bone. Eur J Cancer England.

[26] Osaka E, Okamura Y, Yoshida Y, Sugitani M, Tokuhashi Y (2019). Intra-articular ectopic ossification associated with denosumab administration for giant cell tumor of bone with intra-articular pathological fracture. J Orthop Sci Japan.

[27] Yi J, Lee YH, Kim SK, Kim SH, Song H-T, Shin K-H (2018). Response evaluation of giant-cell tumor of bone treated by denosumab: histogram and texture analysis of CT images. J Orthop Sci Japan.

[28] Ueda T, Morioka H, Nishida Y, Kakunaga S, Tsuchiya H, Matsumoto Y (2015). Objective tumor response to denosumab in patients with giant cell tumor of bone: a multicenter phase II trial. Ann Oncol Off J Eur Soc Med Oncol England.

[29] Eisenhauer EA, Therasse P, Bogaerts J, Schwartz LH, Sargent D, Ford R (2009). New response evaluation criteria in solid tumours: revised RECIST guideline (version 1.1). Eur J Cancer England.

[30] Egbert RC, Folsom R, Bell J, Rajani R. Denosumab therapy for giant cell tumor of bone pulmonary metastasis. Case Rep Orthop. 2017;2017:2302597. 10.1155/2017/2302597 PMC5442345 28573059

[31] Palmerini E, Chawla NS, Ferrari S, Sudan M, Picci P, Marchesi E (2017). Denosumab in advanced/unresectable giant-cell tumour of bone (GCTB): for how long?. Eur J Cancer England.

[32] Yamagishi T, Kawashima H, Ogose A, Sasaki T, Hotta T, Inagawa S (2016). Disappearance of giant cells and presence of newly formed bone in the pulmonary metastasis of a sacral giant-cell tumor following denosumab treatment: a case report. Oncol Lett Greece.

[33] Oguro S, Okuda S, Sugiura H, Matsumoto S, Sasaki A, Susa M (2018). Giant cell tumors of the bone: changes in image features after denosumab administration. Magn Reson Med Sci Japan.

[34] Fayad LM, Jacobs MA, Wang X, Carrino JA, Bluemke DA (2012). Musculoskeletal tumors: how to use anatomic, functional, and metabolic MR techniques. Radiology United States.

[35] van der Heijden L, Dijkstra PDS, Blay J-Y, Gelderblom H (2017). Giant cell tumour of bone in the denosumab era. Eur J Cancer England.

[36] Verstraete KL, Lang P (2000). Bone and soft tissue tumors: the role of contrast agents for MR imaging. Eur J Radiol Ireland.

[37] van der Woude HJ, Verstraete KL, Hogendoorn PC, Taminiau AH, Hermans J, Bloem JL (1998). Musculoskeletal tumors: does fast dynamic contrast-enhanced subtraction MR imaging contribute to the characterization?. Radiology United States.

[38] Satcher RL, Ravi V, Wang W-L, Oates S (2017). Postpartum treatment of metastatic recurrent giant cell tumor of capitate bone of wrist. Am J Orthop (belle Mead NJ) United States.

[39] Ross AE, Bojescul JA, Kuklo TR (2005). Giant cell tumor: a case report of recurrence during pregnancy. Spine (Phila Pa 1976) United States.

[40] Bussiere JL, Pyrah I, Boyce R, Branstetter D, Loomis M, Andrews-Cleavenger D (2013). Reproductive toxicity of denosumab in cynomolgus monkeys. Reprod Toxicol United States.

[41] Okamatsu N, Sakai N, Karakawa A, Kouyama N, Sato Y, Inagaki K (2017). Biological effects of anti-RANKL antibody administration in pregnant mice and their newborns. Biochem Biophys Res Commun United States.

[42] Boyce RW, Varela A, Chouinard L, Bussiere JL, Chellman GJ, Ominsky MS (2014). Infant cynomolgus monkeys exposed to denosumab in utero exhibit an osteoclast-poor osteopetrotic-like skeletal phenotype at birth and in the early postnatal period. Bone United States.

[43] Zhang Y, Ilaslan H, Bauer TW. Giant cell tumor of bone: imaging and histology changes after denosumab treatment: Comment on: von Borstel D, Taguibao RA, Strle NA, Burns JE. Giant cell tumor of the bone: aggressive case initially treated with denosumab and intralesional surgery. Skelet Radiol Germany. 2017:961–2. 10.1007/s00256-017-2643-4 28389819

[44] Girolami I, Mancini I, Simoni A, Baldi GG, Simi L, Campanacci D (2016). Denosumab treated giant cell tumour of bone: a morphological, immunohistochemical and molecular analysis of a series. J Clin Pathol England.

[45] Akaike K, Suehara Y, Takagi T, Kaneko K, Saito T (2014). An eggshell-like mineralized recurrent lesion in the popliteal region after treatment of giant cell tumor of the bone with denosumab. Skelet Radiol Germany.

[46] Park S-Y, Lee MH, Lee JS, Song JS, Chung HW (2014). Ossified soft tissue recurrence of giant cell tumor of the bone: four case reports with follow-up radiographs, CT, ultrasound, and MR images. Skelet Radiol Germany.

[47] Cooper KL, Beabout JW, Dahlin DC (1984). Giant cell tumor: ossification in soft-tissue implants. Radiology United States.

[48] Ehara S, Nishida J, Abe M, Kawata Y, Saitoh H, Kattapuram SV (1992). Ossified soft tissue recurrence of giant cell tumor of bone. Clin Imaging United States.

[49] Teot LA, O’Keefe RJ, Rosier RN, O’Connell JX, Fox EJ, Hicks DG (1996). Extraosseous primary and recurrent giant cell tumors: transforming growth factor-beta1 and -beta2 expression may explain metaplastic bone formation. Hum Pathol United States.

[50] Bertoni F, Bacchini P, Staals EL (2003). Malignancy in giant cell tumor of bone. Cancer United States.

[51] Domovitov S V, Healey JH. Primary malignant giant-cell tumor of bone has high survival rate. Ann Surg Oncol. United States; 2010;17:694–701. 10.1245/s10434-009-0803-z 19902306

[52] Aponte-Tinao LA, Piuzzi NS, Roitman P, Farfalli GL (2015). A high-grade sarcoma arising in a patient with recurrent benign giant cell tumor of the proximal tibia while receiving treatment with denosumab. Clin Orthop Relat Res United States.

[53] Tsukamoto S, Righi A, Vanel D, Honoki K, Donati DM, Errani C (2017). Development of high-grade osteosarcoma in a patient with recurrent giant cell tumor of the ischium while receiving treatment with denosumab. Jpn J Clin Oncol England.

[54] Palmerini E, Picci P, Reichardt P, Downey G (2019). Malignancy in giant cell tumor of bone: a review of the literature. Technol Cancer Res Treat United States.

[55] Grote HJ, Braun M, Kalinski T, Pomjanski N, Back W, Bleyl U (2004). Spontaneous malignant transformation of conventional giant cell tumor. Skelet Radiol Germany.

[56] Wojcik J, Rosenberg AE, Bredella MA, Choy E, Hornicek FJ, Nielsen GP (2016). Denosumab-treated giant cell tumor of bone exhibits morphologic overlap with malignant giant cell tumor of bone. Am J Surg Pathol United States.

[57] Wu C-C, Hsieh P-P (2018). Denosumab-treated giant cell tumor of the bone mimicking low-grade central osteosarcoma. J Pathol Transl Med Korea (South).

